# Sequential management of traumatic breast hematoma with an anatomically evident flail segment: a case report

**DOI:** 10.1093/jscr/rjag959

**Published:** 2026-10-22

**Authors:** Yoshitaka Ooya, Shuji Takahira

**Affiliations:** Departments of Emergency and Acute Medicine, Saitama Medical University International Medical Center, 1397-1 Yamane, Hidaka City, Saitama Prefecture, Japan; Department of Emergency Medicine, Saitama Medical University Hospital, 38 Moro-Hongo, Moroyama-machi, Iruma-gun, Saitama Prefecture, Japan

**Keywords:** traumatic breast hematoma, flail chest, transcatheter arterial embolization, surgical stabilization of rib fractures, computed tomography

## Abstract

Traumatic breast hematoma is an uncommon injury following blunt chest trauma, but associated chest wall injuries may complicate treatment planning. We report a woman in her 70s who sustained a frontal motor vehicle collision. Contrast-enhanced computed tomography (CT) demonstrated a massive right breast hematoma with active extravasation from a branch of the internal mammary artery and an anatomically evident flail segment involving the right third through fifth ribs. Emergency transcatheter arterial embolization achieved complete hemostasis. Because preoperative CT suggested that the hematoma temporarily stabilized the injured chest wall, hematoma evacuation, and possible surgical stabilization of rib fractures (SSRF) were planned through a single inframammary incision. After hematoma evacuation, manual compression demonstrated abnormal mobility of the flail segment, confirming mechanical chest wall instability, and SSRF was completed through the same incision. Careful CT assessment and intraoperative evaluation may facilitate surgical planning in patients with combined breast and chest wall trauma.

## Introduction

Traumatic breast hematoma is an uncommon injury following blunt chest trauma and is usually managed conservatively. However, active bleeding or progressive hematoma enlargement may require transcatheter arterial embolization (TAE) or surgery [1–6]. In patients with multiple rib fractures, a massive breast hematoma may provide temporary soft-tissue support and make chest wall instability less apparent. We report a case in which computed tomography (CT) findings and intraoperative assessment guided sequential management of a traumatic breast hematoma associated with an anatomically evident flail segment.

## Presentation of case

A woman in her 70s was transferred to our emergency and critical care center after a frontal motor vehicle collision. On arrival, she was alert with a blood pressure of 145/91 mmHg, heart rate of 61 beats/min, respiratory rate of 23 breaths/min, and oxygen saturation of 100% on 10 L/min oxygen via face mask. Physical examination revealed marked swelling and deformity of the right breast without apparent paradoxical chest wall movement. A seatbelt mark was present across the lower abdomen (Fig. 1a). Chest radiography demonstrated extensive soft tissue swelling of the right chest wall (Fig. 1b).

**Figure 1:**
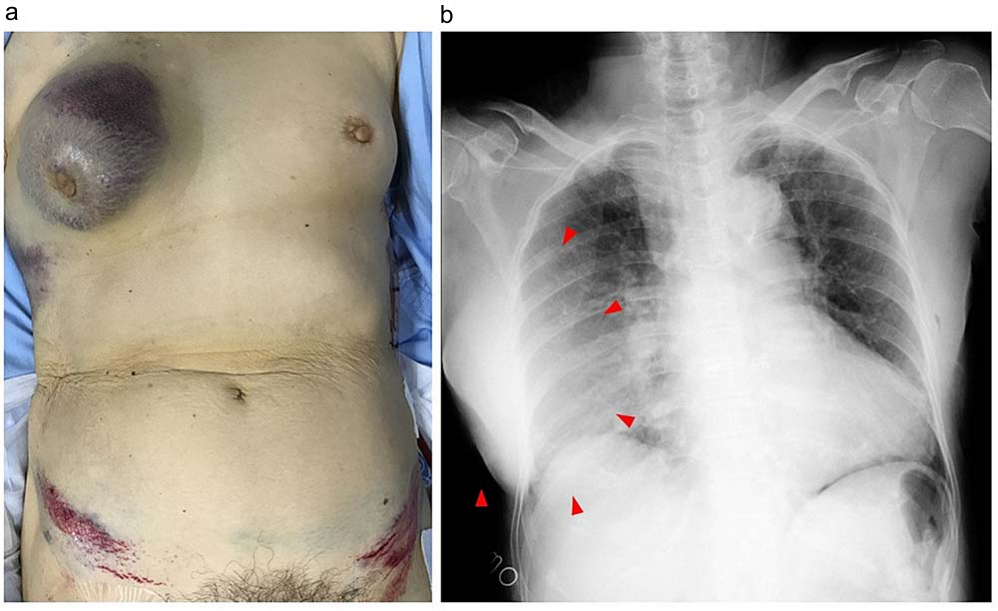
Initial clinical and radiographic findings. (a) Clinical photograph obtained on arrival demonstrating marked swelling and ecchymosis of the right breast caused by a massive traumatic breast hematoma. A seatbelt mark is visible across the lower abdomen. (b) Chest radiograph demonstrating decreased radiolucency of the right chest wall caused by extensive soft tissue swelling (arrowheads).

Contrast-enhanced CT demonstrated a massive right breast hematoma with active contrast extravasation from a branch of the right internal mammary artery, fractures of the right third through seventh ribs with the third through fifth ribs constituting an anatomically evident flail segment, and a left pneumothorax (Fig. 2a–d). The markedly displaced fractures and chest wall deformity were considered indications for possible surgical stabilization of rib fractures (SSRF), while the large hematoma was considered to provide temporary soft-tissue support. A left chest tube was inserted before surgery to treat the pneumothorax and reduce the risk of tension pneumothorax during positive-pressure ventilation.

**Figure 2:**
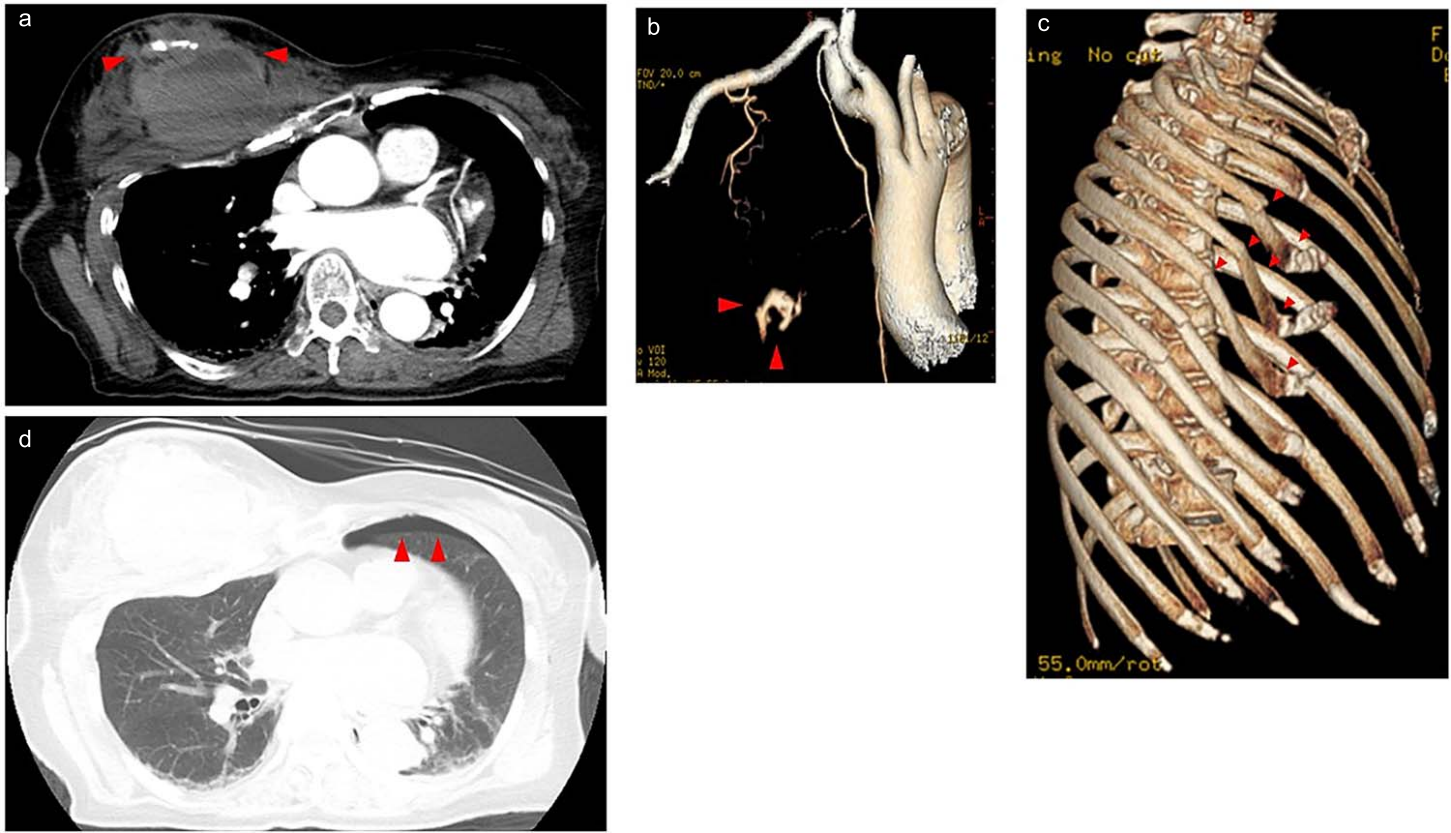
Contrast-enhanced CT before treatment. (a) Axial contrast-enhanced CT demonstrating a massive right breast hematoma with active contrast extravasation (arrow). (b) Three-dimensional CT angiography demonstrating contrast extravasation from a branch of the right internal mammary artery (arrow). (c) Three-dimensional CT reconstruction demonstrating fractures of the right third through seventh ribs, with the third through fifth ribs constituting an anatomically evident flail segment (arrowheads). (d) Axial lung-window CT demonstrating a left pneumothorax (arrowhead).

Emergency TAE using 50% N-butyl-2-cyanoacrylate achieved complete hemostasis (Fig. 3a). After hemodynamic stabilization, surgery was performed the following day. An inframammary incision was planned to allow hematoma evacuation and immediate SSRF if mechanical instability was confirmed, without an additional incision.

**Figure 3:**
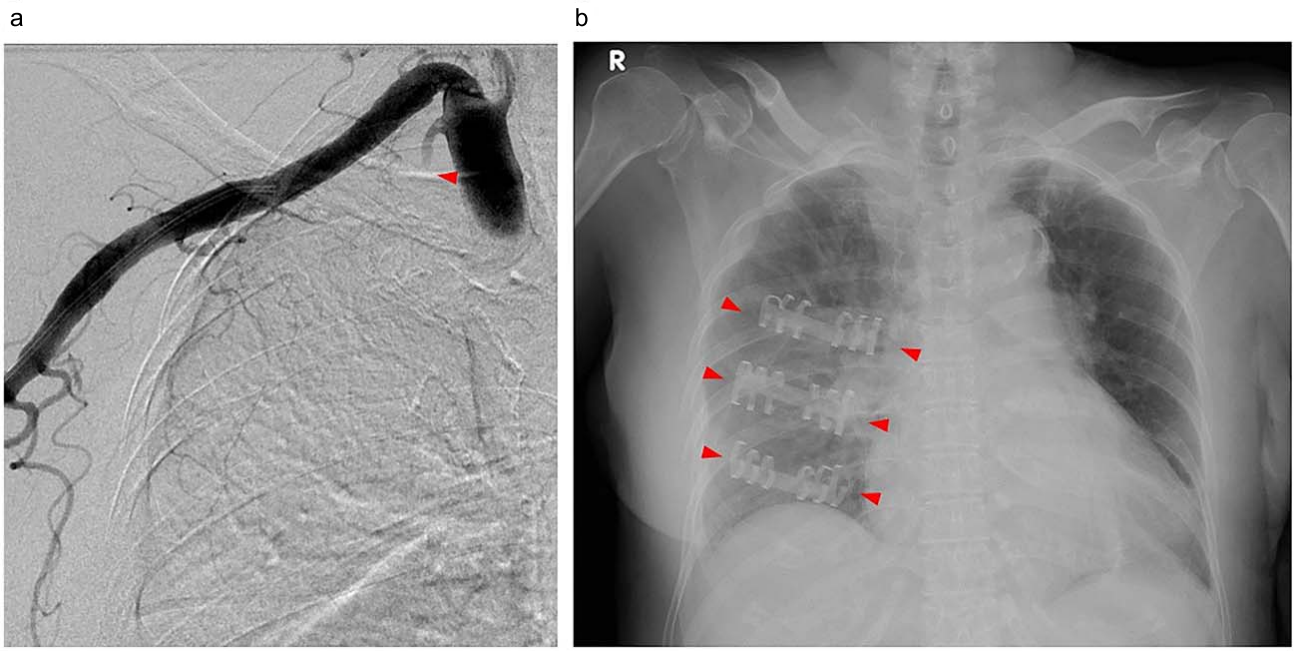
Endovascular treatment and postoperative findings. (a) Selective angiography after transcatheter arterial embolization with N-butyl-2-cyanoacrylate demonstrating complete hemostasis without residual contrast extravasation. (b) Postoperative chest radiograph demonstrating surgical stabilization of the right third through fifth ribs using KANI plates (arrowheads).

After hematoma evacuation, while the patient remained under general anesthesia with positive-pressure ventilation, manual compression of the injured chest wall demonstrated abnormal mobility of the flail segment, confirming mechanical chest wall instability. SSRF of the right third through fifth ribs was therefore performed through the same incision using KANI claw-type titanium plates (24 mm, six-claw; Medical Next, Japan) (Fig. 3b). The sixth and seventh ribs were not fixed because they were less accessible and contributed less to residual instability. The operation lasted 1 h 45 min, and estimated total blood loss, including the evacuated intramammary hematoma, was 423 mL. The postoperative course was uneventful, without rebleeding, breast ischemia, wound infection, or respiratory complications, and the patient was transferred to a rehabilitation facility on hospital day 21.

## Discussion

Clinically significant traumatic breast hematoma is uncommon. Although most traumatic breast hematomas are managed conservatively, active arterial bleeding may require TAE or surgery. Previous reports have demonstrated the effectiveness of TAE for hemorrhage control, but have focused primarily on hemostasis rather than associated chest wall injuries or subsequent operative managemen [4–6].

In this case, CT demonstrated markedly displaced rib fractures and an anatomically evident flail segment despite the absence of apparent paradoxical motion on initial examination. These anatomical findings themselves supported consideration of SSRF. We also considered that the large breast hematoma provided temporary soft-tissue support. Importantly, mechanical instability after evacuation was not identified from spontaneous respiratory motion under positive-pressure ventilation; it was confirmed by abnormal mobility of the flail segment during manual compression. Thus, the indication for SSRF was based primarily on the anatomical severity of the chest wall injury, with anticipated loss of temporary soft-tissue support after hematoma evacuation as an additional consideration.

Current guidelines from the World Society of Emergency Surgery (WSES) and the Chest Wall Injury Society (CWIS) recommend SSRF for appropriately selected patients with flail chest [7–9]. Randomized trials and meta-analyses have shown that SSRF reduces ventilator dependence and pulmonary complications compared with nonoperative management [10–13]. In our patient, preoperative CT enabled the incision and operative sequence to be planned so that hematoma evacuation and SSRF could be completed during a single operation.

This case suggests that a large traumatic breast hematoma may make mechanical instability from severe rib fractures less clinically apparent. CT assessment of fracture displacement and chest wall deformity, together with intraoperative manual assessment, may therefore assist surgical planning. Limitations include the single-case design and the qualitative nature of the assessment. In addition, postoperative CT was not obtained because the postoperative course was uncomplicated, precluding direct CT comparison of thoracic configuration before and after SSRF.

## Conclusion

A massive traumatic breast hematoma may make mechanical instability from an anatomically evident flail segment less clinically apparent. Careful CT assessment and intraoperative manual evaluation may facilitate appropriate surgical planning in patients with combined breast and chest wall trauma.
